# Dendrobium mixture ameliorates type 2 diabetes mellitus with non-alcoholic fatty liver disease through PPAR gamma: An integrated study of bioinformatics analysis and experimental verification

**DOI:** 10.3389/fphar.2023.1112554

**Published:** 2023-02-16

**Authors:** Shuting Zhuang, Jieping Zhang, Xiaohui Lin, Xiaoning Wang, Wenzhen Yu, Hong Shi

**Affiliations:** College of Integrated Traditional Chinese and Western Medicine, Fujian University of Traditional Chinese Medicine, Fuzhou, China

**Keywords:** dendrobium mixture, non-alcoholic fatty liver disease, type 2 diabetes mellitus, network pharmacology, TMT-based quantitative protomics, inflammation, PPAR gamma

## Abstract

Dendrobium mixture (DM) is a patented Chinese herbal medicine indicated which has anti-inflammatory and improved glycolipid metabolism. However, its active ingredients, targets of action, and potential mechanisms are still uncertain. Here, we investigate the role of DM as a prospective modulator of protection against non-alcoholic fatty liver disease (NAFLD) induced by type 2 diabetes mellitus (T2DM) and illustrate the molecular mechanisms potentially involved. The network pharmacology and TMT-based quantitative protomics analysis were conducted to identify potential gene targets of the active ingredients in DM against NAFLD and T2DM. DM was administered to the mice of DM group for 4 weeks, and *db/m* mice (control group) and *db/db* mice (model group) were gavaged by normal saline. DM was also given to Sprague-Dawley (SD) rats, and the serum was subjected to the palmitic acid-induced HepG2 cells with abnormal lipid metabolism. The mechanism of DM protection against T2DM-NAFLD is to improve liver function and pathological morphology by promoting peroxisome proliferator-activated receptor γ (PPARγ) activation, lowering blood glucose, improving insulin resistance (IR), and reducing inflammatory factors. In *db/db* mice, DM reduced RBG, body weight, and serum lipids levels, and significantly alleviated histological damage of liver steatosis and inflammation. It upregulated the PPARγ corresponding to the prediction from the bioinformatics analysis. DM significantly reduced inflammation by activating PPARγ in both *db/db* mice and palmitic acid-induced HepG2 cells.

## 1 Introduction

NAFLD produces two key components of the metabolic syndrome (MetS), glucose and triglycerides (Ferguson and Finck, 2021; Stefan and Cusi, 2022). The association between T2DM and NAFLD is complex and bidirectional, and the two often co-occur in the context of the metabolic syndrome. Consequently, the liver is a key determinant of abnormal glucolipid metabolism. The “twice hit” theory states that NAFLD can further deteriorate and develop into non-alcoholic steatohepatitis (NASH), which mainly manifests as a fatty buildup and inflammation in the liver. Studies have demonstrated that T2DM predisposes to the development of NAFLD through IR and hyperglycemia, and its more severe form, NASH, may contribute to the risk of cirrhosis and malignant tumors of the liver (Jarvis et al., 2020; Targher et al., 2021). The alarming thing is that there are still unapproved drug therapies for the disease. It is particularly crucial to explore effective treatment methods and explain the molecular mechanism underlying T2DM with NAFLD.

Dendrobium mixture (DM) (patent. no. ZL201110408411.0) consisted of Huang Qi [HQ, *Astragalus membranaceus* (Fisch.) Bge.], Shi Hu (SH, *Dendrobium nobile* Lindl.), Dan Shen (DS, Salvia miltiorrhiza Bge.), Niu Xi (NX, *Achyranthes bidentata* Bl.), Wu Wei Zi [WWZ, *Schisandra chinensis* (Turcz.) Baill.], and Zhi Mu (ZM, *Anemarrhena asphodeloides* Bge.), etc. (Chen et al., 2021; Wang et al., 2022; Zheng et al., 2022). Our works here already showed that DM can be advantageous in reducing IR, improving disorders of glucolipid metabolism, anti-oxidation damage, reducing inflammation, and gradually improving diabetic liver and kidney function (Lin et al., 2018; Chen et al., 2021). Therefore, we hope that this study will allow us to explore the targets of action of the active ingredients of dm, as well as to explain some of the mechanisms underlying the treatment of T2DM-NAFLD. We also expect to provide fundamental evidence for future drug research.

Network pharmacology identifies key gene and protein networks associated with target diseases through database information collection and software analysis. Network pharmacology has also gained popularity in studying complex herbal combinations where multiple bioactive components act and interact with multiple genes and protein targets (Hopkins, 2008). Tandem mass tagging (TMTs) is a frequently used technique for differential proteomics and has been widely used for screening disease markers, drug action targeting, animal and plant therapeutic mechanism, animal and plant development and differentiation mechanism, and other fields. Due to their high sensitivity, wide range, high throughput, and high efficiency, TMTs are the application of the most preferred proteomics technology in recent years (Moulder et al., 2018).

The protein changes and potential mechanisms of NAFLD and T2DM after DM intervention will be analyzed by us in this work by TMTs, and then the main active ingredients of DM and their predicted targets of action in NAFLD and T2DM will then be analyzed by network pharmacology theory. We investigated the pharmacological effect of the active ingredients of DM in the therapy of T2DM combined with NAFLD mice based on multiple analyses of bioinformatics.

## 2 Materials and methods

### 2.1 DM preparation

The composition and preparation of DM decoction followed our previous study (Chen et al., 2021; Zheng et al., 2022). All herbs (108 g of the raw materials per day) were first steeped in 800 mL of water and brought to a boil over high heat, then simmered on low heat for 15 min. Filter the liquid and repeat the process with 800 mL of water for the remaining herbs. Finally, the herbal solution from the two decoctions is concentrated in 1.62 g/mL of ingredients in the formula. The main active ingredients in DM included catalpol, harpaside, puerarin, timosaponin BII, astragaloside IV, tanshinone IIA and schisandrin (Zheng et al., 2022).

### 2.2 Animal model and experimental intervention

Ten male *db/m* mice (8 weeks old, 20 ± 2 g), and forty male *db/db* mice (8 weeks old, 36 ± 2 g) from Nanjing Junke Bio-Technology Co., Ltd. The *db/db* mice are Leptin receptor gene deficient mice which have been widely used as a model of diabetes and non-alcoholic fatty liver disease and usually db/m mice were used as the control group for the experiments (Guilbaud et al., 2019; Suriano et al., 2021). The animals were kept in the Animal Experiment Center of Fujian University of Traditional Chinese Medicine. Gavage dosages in DM were determined by consideration of the equivalent conversion of human dose for a mouse model and several studies, including an animal model (Nair and Jacob, 2016). The dosage of Chinese medicine conforms to the standards stipulated in the current Chinese Pharmacopoeia (Cai et al., 2019). The Control group used 10 *db/m* mice, and 20 *db/db* mice, randomly grouped according to blood glucose and body weight, with 10 mice in each group, were used as the model group and the DM group, respectively. All groups were gavaged with normal saline except for the DM group, and the dose of DM group was converted according to the adult clinical equivalent dose, and the DM concentration was 16.2 g/kg/d. The gavage volume of all mice was 0.1 mL/10 g. The gavage was performed at 9:00 a.m. every day, and the body weight was monitored weekly.

After the end of gavage, the blood was taken, fasted without water for 8 h before taking, and 5% uratan was used for intraperitoneal injection; after the mice were anesthetized, the eyes were fully exposed and blood was collected by removing the eyeballs, and blood was gathered in 1.5 mL centrifuge tubes, and place the centrifuge in a low-temperature centrifuge (4°C, 8,000 g, 15 min). Liver tissues were separated and stored partially in 4% paraformaldehyde and partially at −80°C after aspiration of excess liquid. This study obtained approval from the Experimental Animal Ethics Committee of Fujian University of Traditional Chinese Medicine (approval number: FJTCM IACUC2021079).

### 2.3 TMT-based quantitative protomics analysis

#### 2.3.1 Sample preparation with TMT reagents

Liver tissue was lysed in SDT buffer, UA buffer was repeatedly ultrafiltered, and iodoacetamide was added for incubation. Finally, the proteins were digested. A C18 column (Sigma, United States) was used to desalinate the peptides. After protein quantification, sample labeling was performed. Reagents were used iTRAQ reagent (Applied Biosystems) and TMT reagent (Thermo Scientific, United States).

#### 2.3.2 LC-MS/MS analysis

Peptides were coupled to a C18 reversed-phase analytical column (Thermo Scientific Easy Column) for linear gradient separation in buffer and LC-MS/MS analysis using a Q Exactive mass spectrometer (Thermo Scientific) and Easy nLC (Thermo Fisher Scientific).

#### 2.3.3 Bioinformatic analysis

Differential proteins were mapped and annotated by gene ontology (GO) terminology (Blast2GO). The signaling pathways involved in the differential proteins were determined by the Kyoto Encyclopedia of Genes and Genomes (KEGG) database. Functional categories and pathways were considered significant when the *p <* 0.05.

### 2.4 Network pharmacology analysis

#### 2.4.1 Collecting targets for DM for T2DM and NAFLD

The bioactive compounds of the herbal components of DM were analyzed by searching the TCM Systematic Pharmacology Database and Analysis Platform (TCMSP) and the Encyclopedia of Chinese Medicines (ETCM) (Ru et al., 2014; Xu et al., 2019). The two-dimensional structure maps of the selected compounds were obtained from the PubChem database after the gene targets (probability >0.9) of the selected compounds were predicted by the Swiss Target Prediction Database prediction (Daina et al., 2019). Disease genes associated with T2DM-NAFLD were searched using five databases, including the GeneCards database, the Online Mendelian Inheritance in Mandatabase, the therapeutic target database, the DrugBank database, and the DisGeNet database. The search terms were specified as “type 2 diabetes” and “non-alcoholic fatty liver”, and the species was limited to “*Homo sapiens*”. The targets of DM predicted by the databases in T2DM and NAFLD were summarized for the next analysis.

#### 2.4.2 Construction of core subnetwork

**FIGURE 1:**
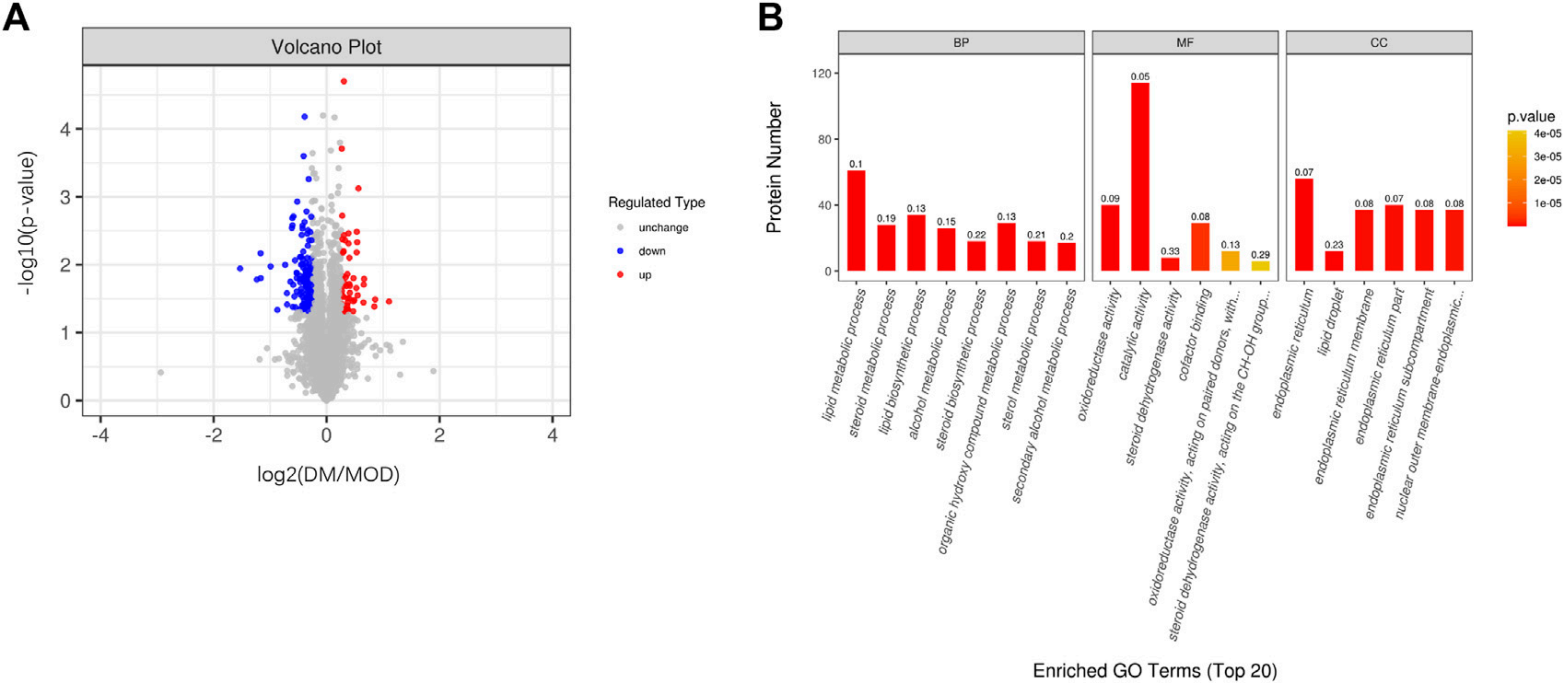
**(A)** Volcano plot showing significantly up- (red) or downregulated (bule) proteins between the DM group and the model group. The gray points are proteins whose expression levels did not change significantly. **(B)** GO terms of the DSEPs in the DM treatment group compared with the model group.

Venny2.1 (https://bioinfogp.cnb.csic.es/tools/venny/) was used to visualize overlapping genes of DM therapeutic targets with disease targets of T2DM and NAFLD. Predicted targets for DM treatment of T2DM-NAFLD were uploaded into the STRING database resulting in protein-protein interaction (PPI) network. To analyze its results even further, the data source of PPI network will be imported into Cytoscape 3.9.1, and the top five molecular targets were screened by CytoHubba plug-in functions to construct the “disease-target-drug” network (Figure 1B).

#### 2.4.3 GO and KEGG pathway enrichment analyses

Therapeutic targets for each bioactive compound in DM were uploaded using the Metascape database, and the results of GO functional enrichment and KEGG pathway enrichment analysis were downloaded for further screening (Zhou et al., 2019).

### 2.5 Glucose and lipid metabolism analysis

Random blood glucose (RBG) levels in the blood samples retrieved from the tail vein of each mouse were measured weekly using the glucose meter (Roche, United States). At the end of the experiment, the serum levels of triglycerides (TG) (Solarbio, China, BC0625), total cholesterol (TC) (Solarbio, China, BC 1985), low-density lipoprotein cholesterol (LDL-C) (Solarbio, China, BC5335), high-density lipoprotein cholesterol (HDL-C) (Solarbio, China, BC5325), aspartate aminotransferase (AST) (Sigma-Aldrich, United States, MAK055) and alanine aminotransferase (ALT) (Sigma-Aldrich, United States, MAK052-1 KT) were measured using commercial kits based on the protocols from the manufacturers.

### 2.6 Liver index

After the mice were sacrificed, the intact liver was taken out and photographed. The liver index was calculated by the following equation:

Liver index=liver weight/body weight×100%

### 2.7 Hematoxylin and eosin (HE) staining

After blood was obtained from the mice, the intact livers were photographed and weighed. Liver tissue was placed in a solution of 4% paraformaldehyde and it was kept at −80°C. The liver was sectioned and the thickness was limited to 5 μm. Xylene and gradient alcohol was used for dewaxing, and hematoxylin and eosin were used for staining and sealed with neutral resin. Neutral resin was used to glue coverslips, which were observed and photographed using a Nikon 55i microscope (Nikon, Japan). The Non-Alcoholic Fatty Liver Activity Score (NAS) was applied in this drug treatment trial (Kleiner et al., 2005). In the NAS scoring system, hepatocellular steatosis was scored 0–3, hepatocellular ballooning was scored 0–2, and inflammation within the liver lobules was scored 0–3.

### 2.8 Cell culture and induction of palmitic acid

HepG2 cells were acquired from the department of biochemistry, Fujian University of Traditional Chinese Medicine. HepG2 cells were cultured in DMEM high sugar medium (Gibco, United States, 11995065) consisting of 10% fetal bovine serum (FBS; Gibco, United States, 10099141) and 1% penicillin-streptomycin (Gibco, United States, 15140163). High glucose medium combined with 250 μM palmitic acid (PA, Solarbio, China, H8780) intervened in the cells for 24 h to induce cellular steatosis.

### 2.9 Preparation and screening of serum containing DM

After 40 male Sprague-Dawley (SD) rats were adaptively fed for 1 week, 15 mice were then randomly selected as the control group and given normal saline, while the rest of the mice were fed DM (10.8 g/kg/d once a day). All the mice were fed for 7 days. On the last day (fasting without water for 12 h), 20% urethane was administered for anesthesia. After blood was drawn by abdominal arter, it was centrifuged at 8,000 g for 15 min. The serum was collected from each mouse and combined within the same group. The serum sample was sterilized in a 56°C water bath, filtered by 0.22-micron filter, and stored at −80°C until analysis. The HepG2 cells were treated with DMEM media only or DMEM media with PA at 250 μM for 24 h. The PA-induced cells were then intervened with diluted serum from normal saline-treated SD rats or DM-treated SD rats, respectively. The cell or cell supernatant was subjected to the following bioassays.

### 2.10 Oil red O staining

Frozen sections of liver tissue from each group and crawling slices of HepG2 cells treated with different serum drugs were fractionated with 60% isopropanol for 10 min; oil red O (Sigma-Aldrich, United States, 0625) was stained for 10 min, washed with PBS, fractionated with 60% isopropanol for 15 s, stained with hematoxylin (Solarbio, China, H8070), blow-dried crawling slices, and glycerol gelatin The films were sealed and photographed for storage. The films were mounted with glycerol gelatin (Beyotime, China, C0187). The films were observed and photographed under the light microscope (Nikon 55i microscope, Japan).

### 2.11 Western blot analysis

RIPA lysis (Beyotime, China, P0013B) buffer containing protease inhibitors was used to lyse liver tissue and HepG2 cells by ultrasonic lysis at low temperature and centrifugation. Protein content determination according to BCA kit instructions (PC0020, Solarbio, China). Proteins were separated by electrophoresis, transferred to PVDF membranes (IPFL00010, Millipore, United States), and blocked by incubation with blot blocking buffer for 10 min (P30500, NCM Biotec, China) at room temperature. The membrane is then placed into a primary antibody box containing anti-PPARγ (Cell Signaling Technology, United States, #2435), anti-PGC-1α (Cell Signaling Technology, United States, #2178), and anti-β-actin (Proteintech, United States, 2D4H5) for binding at 4°C overnight. All primary antibodies were diluted at 1:1000. The membranes were then eluted with TBST and incubated with the secondary antibodies including HRP-conjugated Goat anti-rabbit (Proteintech, America, 10366-1-AP) and HRP-conjugated Goat anti-mouse (Proteintech, America, 3H9D1) for 1 h. The grayscale values of the protein bands were analyzed using Image Lab software, and the ratio of the target protein to the reference protein was calculated and then statistically analyzed.

### 2.12 Measurements of cytokine

Concentrations of serum and HepG2 cells treated with serum drugs of IL1β, IL6, and TNFα were measured by ELISA kit (Abcam, United States, Mouse IL-6 ELISA Kit, ab222503), (Abcam, United States, Human IL-6 ELISA Kit, ab178013), (Abcam, United States, Mouse IL-1β ELISA Kit, ab197742), (Abcam, United States, Human IL-1β ELISA Kit, ab214025), (Abcam, United States, Human TNFα ELISA Kit, ab181421), (Abcam, United States, Mouse TNFα ELISA Kit, ab208348). The serum and cell samples were subjected to the 96-well plates and coupled with antibody cocktails after various treatments and co-incubated for 2 h. After another wash, the TMB Development Solution was incubated for 10 minis. The stop solution was added and the amount was measured by their corresponding calibration curves.

### 2.13 Statistical analysis

Data are shown as mean ± standard deviation. Data conforming to a normal distribution were compared between multiple groups by one-way ANOVA analysis, and the Tukey test was performed between groups when the variances were equal; data not conforming to a normal distribution were compared between groups by a non-parametric test. *p* < 0.05 was considered a statistically significant difference.

## 3 Results

### 3.1 Identification of differentially significant expressed proteins in the liver after dm treatment

In significantly different protein screens, expression fold changes (FC) were compared, and when FC was greater than 1. $\dot{2}$ -fold or less than 0.8 $\dot{3}$ -fold and *p* < 0.05 (*t*-test) was the criterion for significant up- and downregulation of protein quantity between groups. As shown in the volcano plot, blue dots indicate proteins downregulated in the DM group compared to the model group, red represents upregulated proteins, and proteins with no difference are in gray (Figure 1A). The top 15 differentially significant expressed proteins (DSEPs) (up and downregulated) are listed in Tables 1, 2. Dimethylaniline monooxygenase [N-oxide-forming] 3 (*Fmo3*), Farnesyl pyrophosphate synthase (*Fdps*), Cytohesin-3 (*Cyth3*), Kynurenine--oxoglutarate transaminase 3 (*Kyat3*), Isopentenyl-diphosphate Delta-isomerase 1 (*Idi1*), Sulfotransferase 1E1 (*Sult1e1*), etc. showed notable upregulation. Downregulated proteins included vacuolar protein sorting-associated protein 8 homolog (*Vps8*), dual specificity protein phosphatase 12 (*Dusp12*), fascin (*Fscn1*), putative bifunctional UDP-N-acetylglucosamine transferase and deubiquitinase ALG13(*Alg13)*, p53 and DNA damage-regulated protein 1(*Pdrg1*), etc.

**TABLE 1:** Selected significantly upregulated proteins in the Dendrobium mixture (DM) group compared with the model (MOD) group.

Gene name	Protein name	Fold change (DM/MOD)	*p*-Value
Fmo3	Dimethylaniline monooxygenase [N-oxide-forming] 3	2.15177974	0.03486014
Fdps	Farnesyl pyrophosphate synthase	1.81523714	0.03248932
Cyth3	Cytohesin-3	1.79518214	0.04138613
Kyat3	Kynurenine--oxoglutarate transaminase 3	1.58382788	0.01615924
Idi1	Isopentenyl-diphosphate Delta-isomerase 1	1.57733989	0.0196337
Sult1e1	Sulfotransferase 1E1	1.5722069	0.03604566
Got1	Aspartate aminotransferase, cytoplasmic	1.4780465	0.0007515
Mgst3	Microsomal glutathione S-transferase 3	1.46398517	0.02826162
*C*th	Cystathionine gamma-lyase	1.45567752	0.00466174
Ass1	Argininosuccinate synthase	1.44866073	0.00327519
Agxt	Serine--pyruvate aminotransferase, mitochondrial	1.44621756	0.00657333
Aldh1l1	Cytosolic 10-formyltetrahydrofolate dehydrogenase	1.44055477	0.02204724
Acly	ATP-citrate synthase	1.40603532	0.03317841
Sult1a1	Sulfotransferase 1A1	1.39086313	0.01578916
Hmgn2	Non-histone chromosomal protein HMG-17	1.38870532	0.03481204

**TABLE 2:** Selected significantly downregulated proteins in the Dendrobium mixture (DM) group compared with the model (MOD) group.

Gene name	Protein name	Fold change (DM/MOD)	*p*-Value
Vps8	Vacuolar protein sorting-associated protein 8 homolog	0.83332451	0.02896317
Dusp12	Dual specificity protein phosphatase 12	0.83275542	0.03357374
Fscn1	Fascin	0.83055614	0.00436564
Alg13	Putative bifunctional UDP-N-acetylglucosamine transferase and deubiquitinase ALG13	0.83048436	0.01074288
Tmem205	Transmembrane protein 205	0.82983434	0.00795468
Acp2	Lysosomal acid phosphatase	0.8296204	0.01669829
Pdrg1	p53 and DNA damage-regulated protein 1	0.82799098	0.00195912
Prkci	Protein kinase C iota type	0.82685673	0.02009944
Celf2	CUGBP Elav-like family member 2	0.82614131	0.04124738
Acot1	Acyl-coenzyme A thioesterase 1	0.82596656	0.01460239
Arap1	Arf-GAP with Rho-GAP domain, ANK repeat and PH domain-containing protein 1	0.82455461	0.02439473
Pros1	Vitamin K-dependent protein S	0.82229932	0.02561268
Tex2	Testis-expressed protein 2	0.82113709	0.01997845
Ces1f	Carboxylesterase 1F	0.82083517	0.01359083
Gpat4	Glycerol-3-phosphate acyltransferase 4	0.81824183	0.00331068

FC (Fold Change) refers to the multiple of the difference in the expression of the same protein between two samples; FC > 1. $\dot{2}$ indicates upregulated proteins, and FC < 0.8 $\dot{3}$ indicates downregulated proteins.

### 3.2 GO functional enrichment analyses of TMTs

Here, we show the top 20 results of GO analysis (Figure 1B). Lipid metabolism and biosynthetic processes, steroid metabolism and biosynthetic processes, alcohol metabolism processes, and sterol metabolism processes were significantly enriched in BP terms after DM intervention. In the MF group, oxidoreductase activity, catalytic activity, and steroid dehydrogenase activity were significantly enriched. In the CC term, we found that the endoplasmic reticulum was remarkably enriched. These results suggest that DM administration resulted in a greater enrichment of lipid metabolic processes and oxidative stress in the endoplasmic reticulum.

### 3.3 KEGG pathway enrichment analysis of TMTs

**FIGURE 2:**
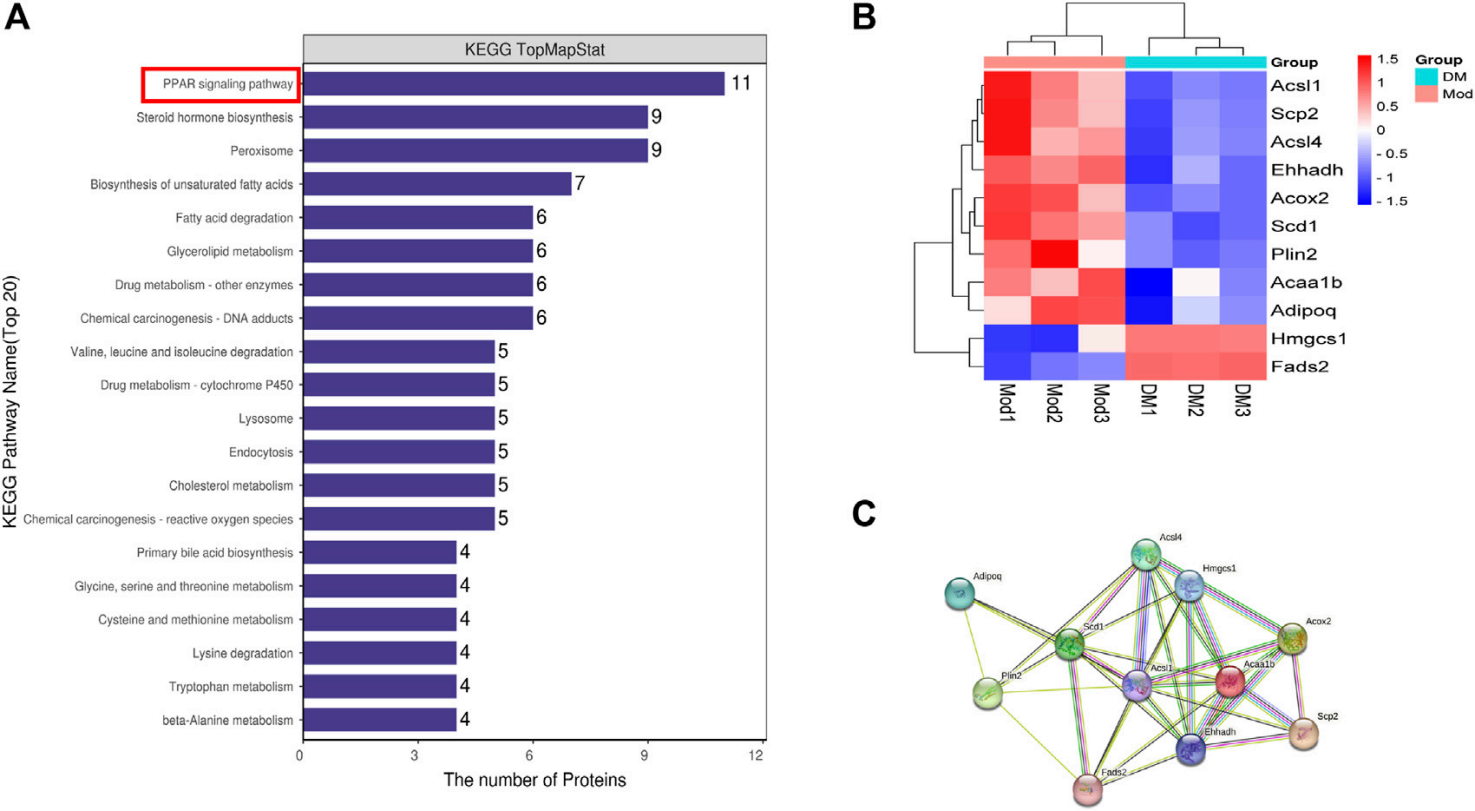
**(A)** The top 20 KEGG pathways enriched in DSEPs. **(B)** Heat map of differentially expressed genes in peroxisome proliferators-activated receptor (PPAR) signaling pathway. **(C)** Search tool for recurring instances of neighboring genes (STRING) network visualization of the genes in differentially expressed genes in the PPAR signaling pathway. Edges represent protein-protein associations.

DSEPs after DM intervention in *db/db* mice were uploaded to KEGG pathway enrichment analysis, thus exploring potential pathways for DM intervention. Here, we show the 20 pathways with the highest enrichment (Figure 2A). We performed a preliminary classification of these results, in which five pathways related to amino acid metabolism, including valine, leucine and isoleucine degradation, tryptophan metabolism, lysine degradation, cysteine and methionine metabolism, glycine and serine and threonine metabolism. The pathway related to lipid metabolism consists of steroid hormone biosynthesis, biosynthesis of unsaturated fatty acids, glycerolipid metabolism, fatty acid degradation, and primary bile acid biosynthesis. The pathway related to transport and catabolism consists of peroxisome, endocytosis, and lysosome. The related pathway of Xenobiotics biodegradation and metabolism include cytochrome P450 and Drug metabolism - other enzymes. The beta-Alanine metabolism belongs to Metabolism. The cholesterol metabolism belongs to the digestive system. A PPAR signaling pathway is part of the endocrine system, which involves the most abundant protein changes. According to our results, after DM intervention, a total of 11 genes on the PPAR pathway were significantly altered, including lipid biosynthesis (*Scd1*), lipid degradation (*Adipoq*), lipid storage (*Plin2*), lipid transport (*Scp2*), lipid synthesis (*Fads2*), β-oxidation (*Acsl1, Acsl4, Acox2, Ehhadh)*, liposynthesisand cholesterol metabolism (*Hmgcs1*) (Figure 2B). In the PPAR signaling pathway, the *Hmgcs1* and *Fads2* genes were upregulated, and the *Scp2, Ehhadh, Acsl1, Acaa1b, Acox2, Plin2, Scd1, Acsl4,* and *Adipoq* genes were downregulated, all of which were closely related to lipid metabolism. Furthermore, STRING analysis was used to visualize the interaction network of significantly altered genes (Figure 2C).

### 3.4 Therapeutic target genes of DM in T2DM and NAFLD

**FIGURE 3:**
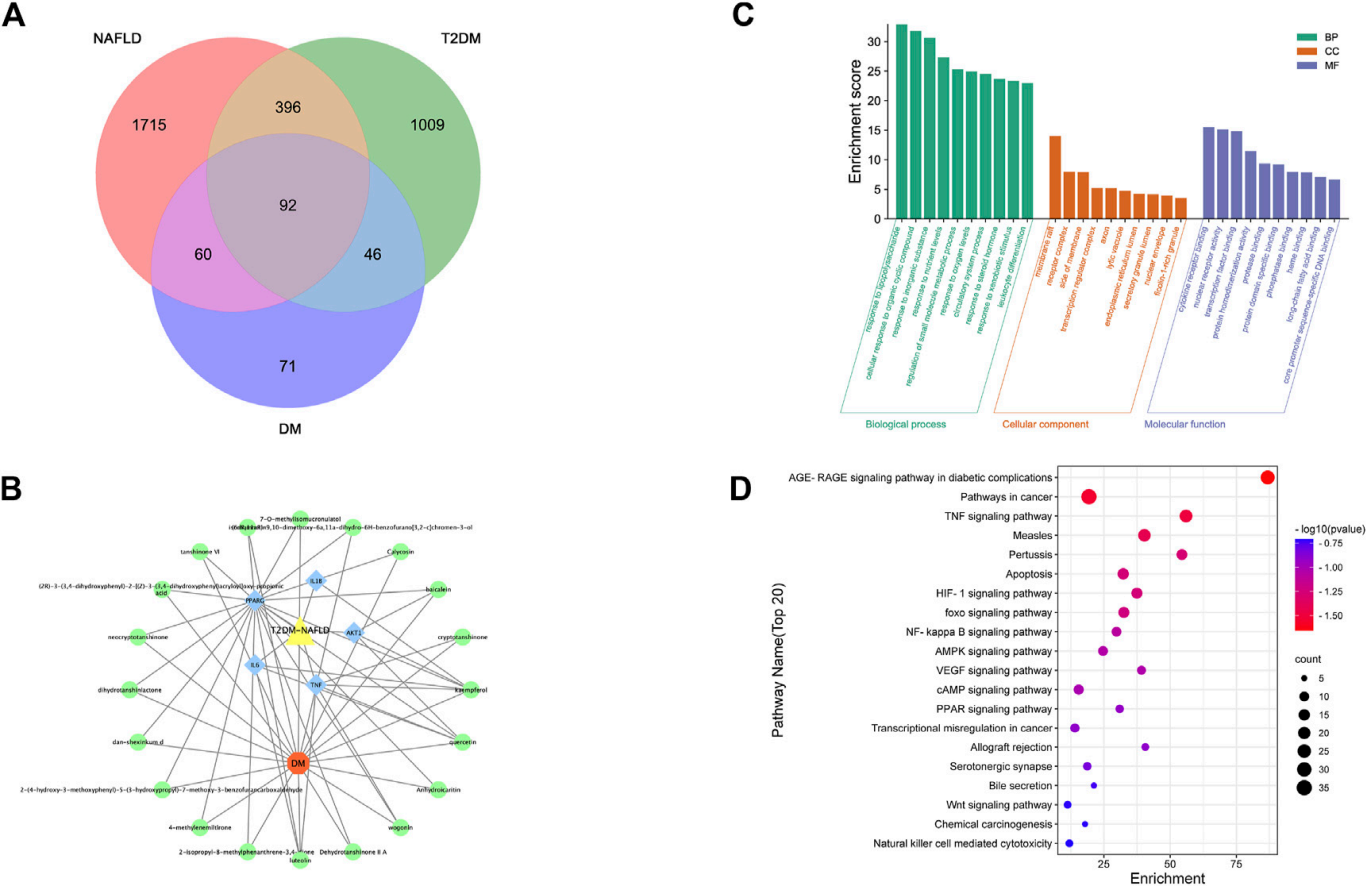
**(A)** Venn of the gene targets of DM, T2DM and NAFLD; **(B)** Network of “drug-gene-disease”. The Orange hexagon represents DM, and the green circles represent components of the DM. The blue square highlighted the gene targets with high degrees. The yellow triangle represents two diseases of NAFLD and T2DM;**(C)** Top 20 GO terms relevant to the action of DM against NAFLD and T2DM. **(D)** Top 20 KEGG relevant to the action of DM against NAFLD and T2DM. The count of each KEGG pathway is indicated by the circle size, and the -log10 *p*-value was indicated by the color.

We acquired 1,543 disease genes for T2DM and 2,263 for NAFLD. The intersection of the target genes in T2DM and NAFLD was then mapped. The Venn diagram revealed 92 overlapping therapeutic targets of T2DM and NAFLD (Figure 3A). Through the TCMSP, ETCM, and literature databases, 115 main active compounds were identified as key drug-like components from six ingredients of DM (Shi Hu: *n* = 9, Wu Wei Zi: *n* = 8, Huang Qi: *n* = 16, Dan Shen, *n* = 56, Niu Xi: *n* = 15, Zhi Mu: *n* = 11). Eventually, there were 271 total gene targets associated with the main active compounds (Supplementary Material S1). The protein-protein interaction network drawn from the STRING database indicated that the proteins encoded by these target genes have complicated associations.

### 3.5 PPI network and core subnetwork

The PPI network was imported into Cytoscape for further analysis. The final five key genes were screened after two filtrations using CytoHubba to construct the interactive network of “drug-gene target-disease” (Figure 3B).

### 3.6 GO functional enrichment and KEGG pathway enrichment analysis of therapeutic target genes

The 92 overlapping genes were mapped for enrichment analysis of the GO terms and KEGG pathways. The results of GO enrichment analysis of DM including molecular function, cellular components, and biological processes are shown in Figure 3C. The top 20 KEGG pathways that may play the most significant role in the mechanisms action of DM are shown in Figure 3D. Majority of these pathways were associated with inflammation and metabolism.

### 3.7 DM regulated glycolipid metabolism in T2DM-NAFLD mice

**FIGURE 4:**
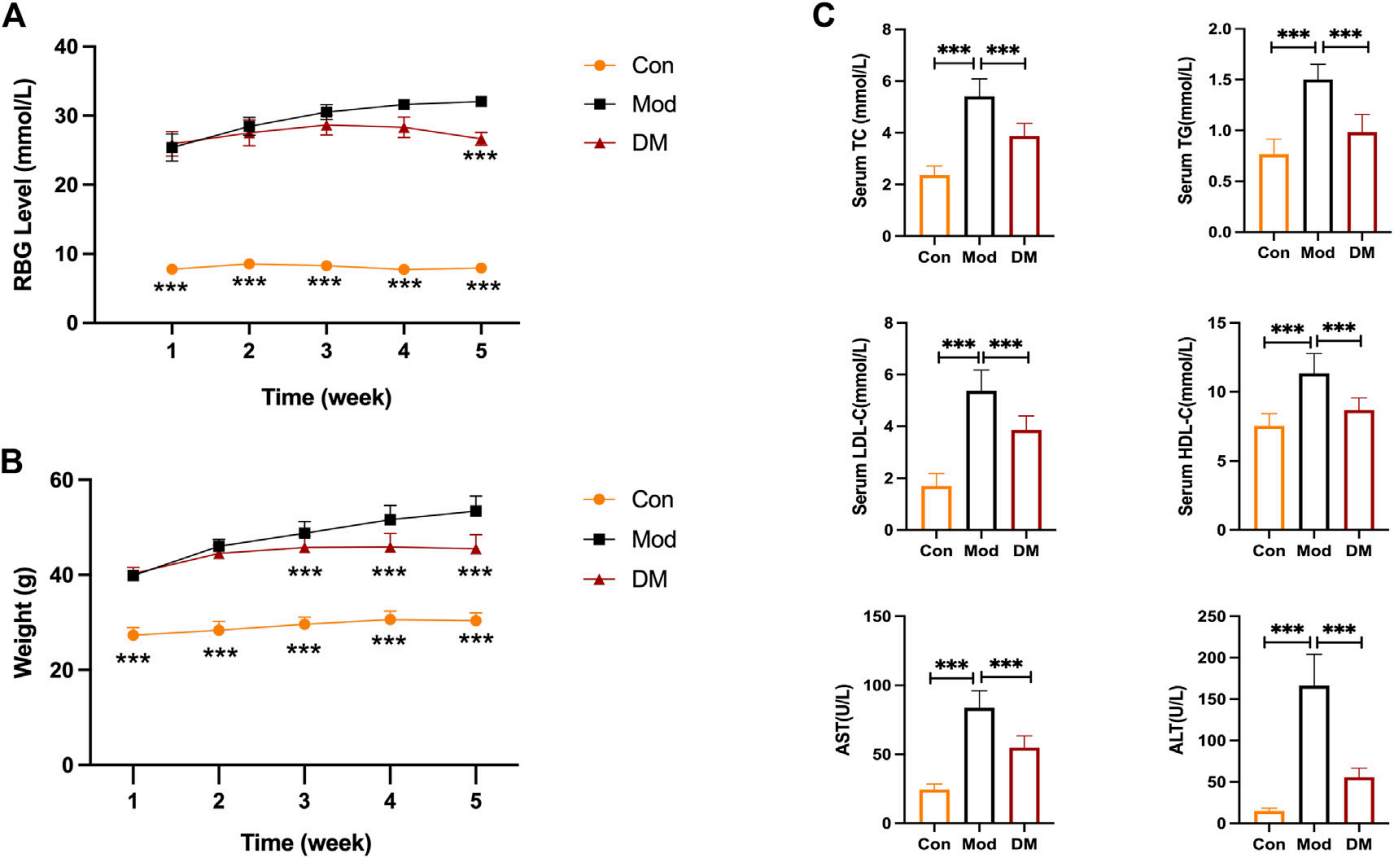
**(A)** DM reduced the elevated RBG level (mmol/L) in *db/db* mice (*n* = 10 per group); **(B)** DM reduced the body weight (g) in *db/db* mice (*n* = 10 per group). ***p < 0.001 vs. Mod as assessed by one-way ANOVA with Tukey’s multiple comparison test;**(C)** The serum levels of TC (mmol/L), TG (mmol/L), HDL-C (mmol/L), LDL-C (mmol/L), AST (U/L) and ALT (U/L) I n the *db/db* mice (*n* = 10 mice per group). ***p < 0.001 vs. Mod group as assessed by one-way ANOVA with Tukey’s multiple comparison test; Con: control group; Mod: model group; DM: DM treatment group.

The random blood glucose (RBG) in *db/db* mice gradually increased to >25 mmol/L with age (Figure 4A). It is to be noted that due to the limitation of the glucose meter, when the blood glucose of the mice exceeded the maximum value of the glucose meter (33.3 mmol/L), it was recorded as 33.3 mmol/L. Compared with the model group, RBG showed a decreasing trend after DM administration for 2 weeks, a significant decrease after DM administration for 3 weeks, and similar results in the fourth week. In Figure 4B, the body weight in the model group (*db/db* mice, 39.9 ± 1.20 g) before treatment was greatly (*p <* 0.001) higher than the control group (*db/m* mice, 27.30 ± 1.64 g). The body weight in the DM treatment group (40.3 ± 1.26 g) was comparable to the model group before treatment. However, at week 4, the body weight in the DM group reduced (45.5 ± 3.0 g) lower than the model group (53.40 ± 3.17 g) (*p* < 0.001). The serum levels of TC (5.41 ± 0.67 mmol/L vs. 2.36 ± 0.35 mmol/L), TG (2.70 ± 0.15 mmol/L vs. 0.83 ± 0.13 mmol/L), HDL (11.34 ± 1.45 mmol/L vs. 7.52 ± 0.88 mmol/L) and LDL (5.37 ± 0.81 mmol/L vs. 1.70 ± 0.49 mmol/L), AST (83.70 ± 12.41 U/L vs. 24.56 ± 3.92 U/L), ALT (166.40 ± 37.79 U/L vs. 15.09 ± 3.56 mmol/L) in the model group were all obviously higher than the control group (*p* < 0.001) (Figure 4C). After DM treatment, the serum levels of TC (3.88 ± 0.49 mmol/L), TG (1.44 ± 0.29 mmol/L), HDL (8.67 ± 0.88 mmol/L) and LDL (3.87 ± 0.55 mmol/L), AST (54.93 ± 8.46 U/L), ALT (55.93 ± 10.70 U/L) were reduced through comparing to the model group (*p* < 0.001).

### 3.8 DM attenuated liver damage in T2DM-NAFLD mice

**FIGURE 5:**
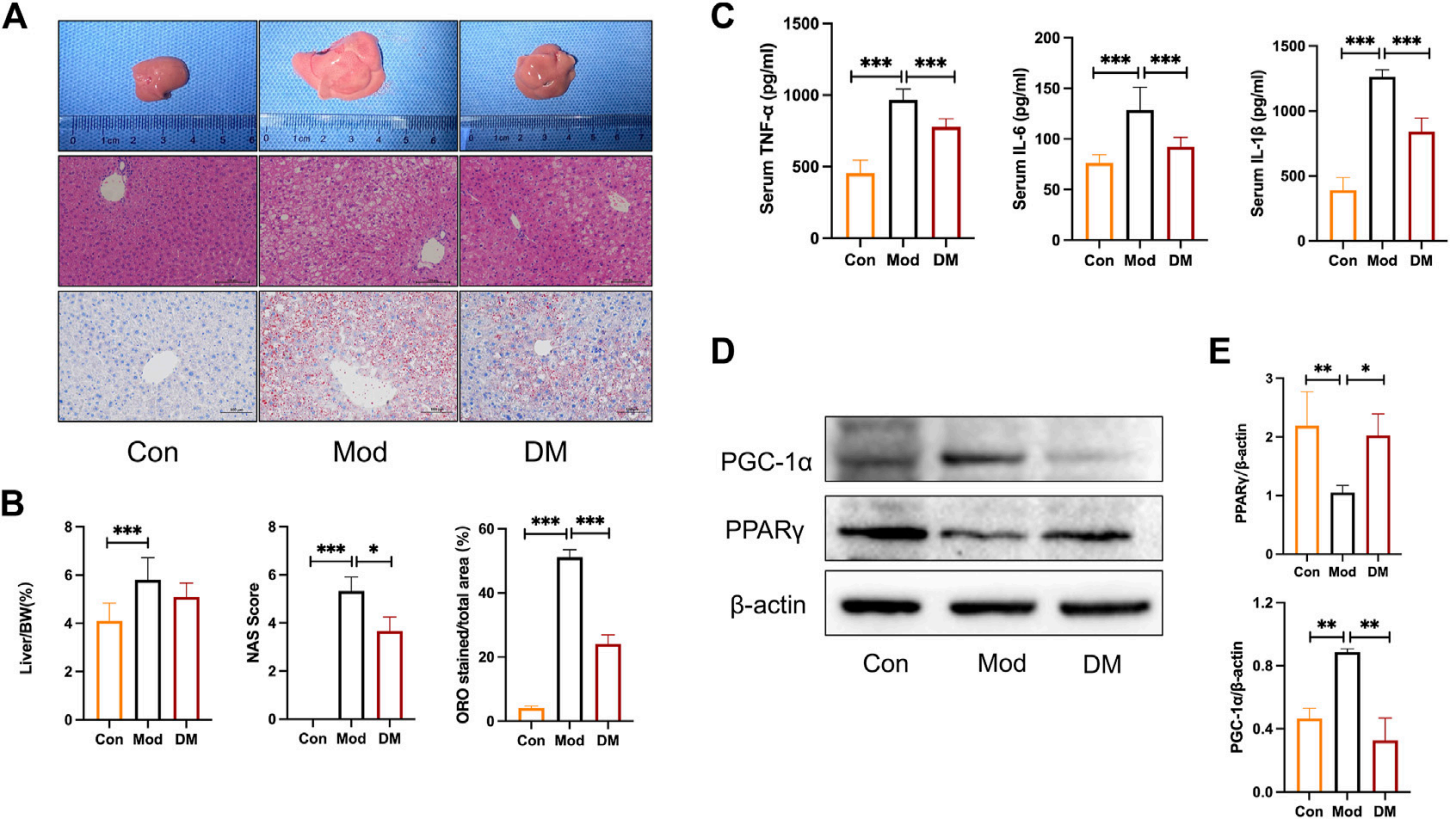
**(A)** Effect of DM on morphology and histopathologic changes in the liver. Representative sections of the liver specimen, H&E staining (scale bars = 100 μm); **(B)** DM reduced the liver weight/BW, NAS, and lipid droplets percentage (%) in the liver. *p < 0.05 and***p < 0.001 vs. Mod group as assessed by one-way ANOVA with Tukey’s multiple comparison test. **(C)** The effect of DM on serum TNF-α, IL-1β, and IL-6 (pg/mL), ***p < 0.001 vs. Mod group as assessed by one-way ANOVA with Tukey’s multiple comparison test. **(D)** Western blot images (*n* = 3 individual experiments); **(E)** their statistical analysis of protein expressions of PPARγand PGC-1α in liver tissue. *p < 0.05 and**p < 0.01 vs. Mod group as assessed by one-way ANOVA with Tukey’s multiple comparison test.

Macroscopical differences were observed in the liver between the control and model group, with the liver in the model group manifested as hepatomegaly (Figure 5A). Hematoxylin staining of the livers of control mice showed that the liver tissue was structurally intact, with neatly arranged hepatocytes of uniform size and no obviously fatty degeneration. In contrast, *db/db* mice clearly showed significant lipoatrophy. The liver index showed that the liver in the model group was more higher than that in the control group (*p* < 0.001). NAS analysis also showed that the model group was significantly higher than that in the control group and was attenuated by DM (*p* < 0.05). In addition, the percentage of hepatocyte lipid accumulation was significantly higher in *db/db* mice (51.13% ± 2.35%, *p* < 0.001) and significantly attenuated by DM (24.8% ± 2.84%, *p* < 0.001).

### 3.9 DM upregulated PPARγ, reduced inflammation in T2DM-NAFLD mice

As shown in Figure 5C, the inflammatory cytokines IL-1β, TNF-α, and IL-6 were all increased greatly in model group (*p* < 0.001), whereas DM significantly inhibited their productions (*p* < 0.001). These results noticed that the effect of DM was associated with an inhibited susceptibility to inflammations. As suggested by the network pharmacology and TMT-based quantitative protomics analysis that intracellular signaling pathways related to PPAR were involved in the pharmacological actions of DM, experimental verification was conducted on the diabetic mice. In the diabetic group, we observed significantly reduced PPARγ (*p* < 0.01) and induced PGC-1α (*p*<0.01) (Figure 5D). However, the intervention of DM significantly increased PPARγ expressions (*p* < 0.05) and reduced significantly higher levels of PGC-1α (*p*<0.01), which corresponded to the predicted action of DM in the network pharmacology and TMT-based quantitative protomics analysis.

### 3.10 Serum containing DM regulated the steatosis in PA-induced HepG2 cells

**FIGURE 6:**
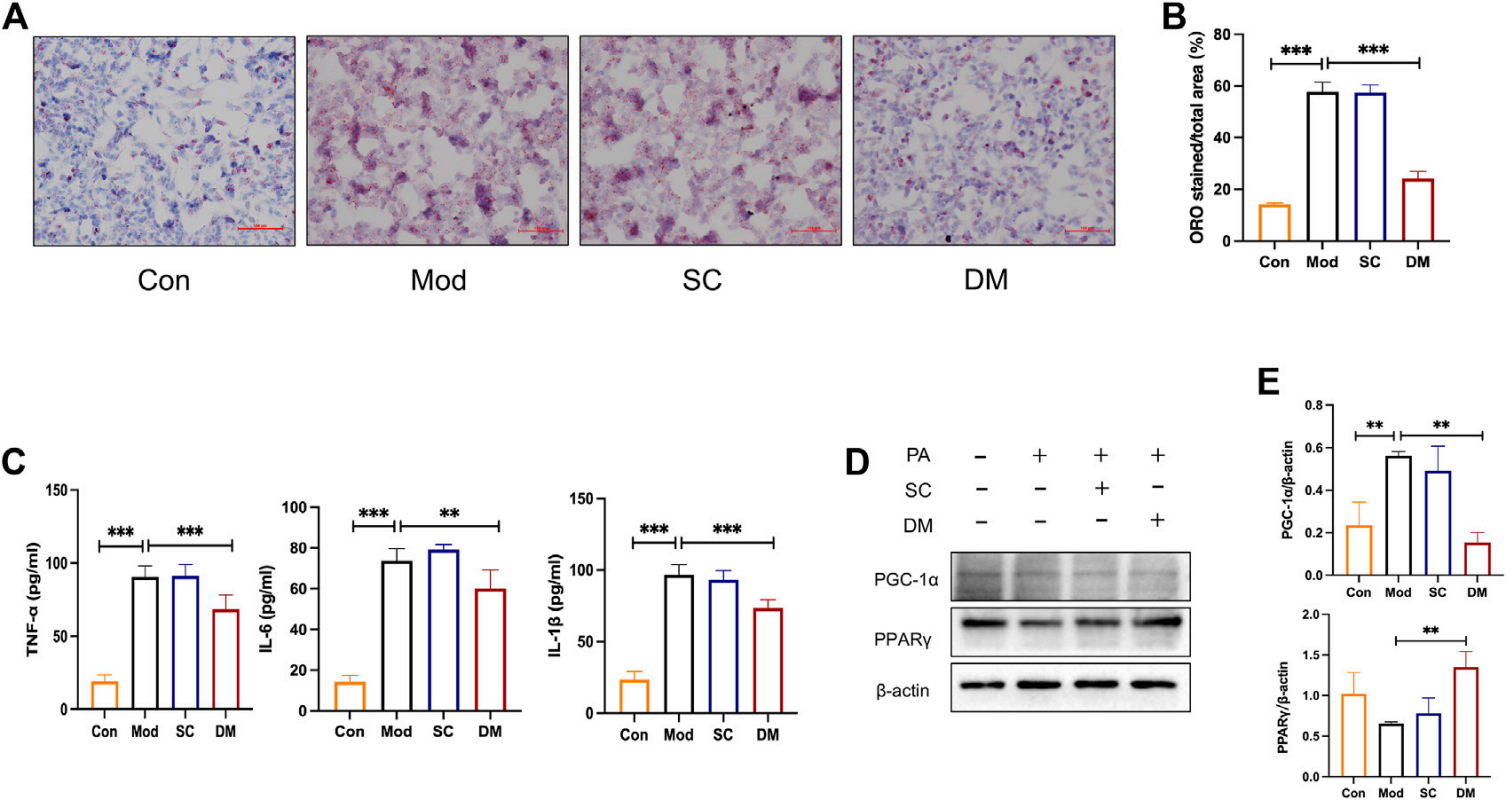
**(A)** Oil Red O staining for HepG2 cells; **(B)** quantitative analysis of lipid. DM reduced the lipid accumulation in HepG2 cells induced by the 24 h coincubation of PA 250 μM. Visual observation of lipid content was captured by microscope (×200): the control cells treated with only media (Con), cells treated with PA (250 μM) for 24 h (Mod), cells pretreated with PA 250 μM for 24 h and then cultured with serum from mice administered with saline only (diluted to 2.5%) for 24 h (Serum Control, SC), and cells pretreated with PA 250 μM for 24 h and then cultured with serum DM for 24 h (DM). The quantitative analysis of cellular steatosis was measured through deposited Oil Red O in the cells. Statistical significance was determined by one-way ANOVA and the values are mean ± STD. ***p < 0. 001 vs. Mod group. **(C)** The effect of cells TNF-α, IL-1β and IL-6 (pg/mL), **p < 0. 01 and ***p < 0.001 vs. Mod group as assessed by one-way ANOVA with Tukey’s multiple comparison test. **(D)** Western blot images (*n* = 3 individual experiments); **(E)** their statistical analysis of protein expressions of PPARγand PGC-1α in HepG2 cells. **p < 0.01 vs. Mod group as assessed by one-way ANOVA with Tukey’s multiple comparison test.

Oil red o staining was used to evaluate the degree of lipid accumulation in Hepatocyte with or without DM treatment. As shown in Figure 6A, the co-incubation of PA and SC groups for 24 h induced an obvious intracellular lipid accumulation as evidenced by the strong red staining. The statistical analysis in Figure 6A demonstrated that the lipid droplet percentage was approximately 3 times that of the control group (*p* < 0.001). However, the lipid droplets in the DM group were significantly lower than that in the model group (*p* < 0.001) (Figure 6B).

### 3.11 Mechanisms of action of DM by integrating bioinformatics analysis and experimental validation in HepG2 cells

To test the expression of inflammation, cytokine analysis was performed using Elisa kit measured HepG2 cells (Figure 6C). After PA induced, the levels of IL-1, TNF, and IL-6 were greatly higher in the model and serum control groups (*p* < 0.001), showing that PA promoted the secretion of cellular inflammatory factors and that saline was not significantly effective. In contrast, inflammation levels in the DM group were significantly lower than those in the mod group (*p* < 0.001or *p* < 0.01). The mechanisms action of DM was investigated based on the bioinformatic analysis that the action of DM was relevant to the PPAR pathway. To further investigate the mechanisms of action of serum DM, Western blot analysis was performed to examine the level of PPARγ as guided by the bioinformatic analysis. In Figure 6D, PA increased the level of PGC-1α (*p*<0.01). With the effect of saline being insignificant, the serum containing DM significantly induced significantly higher levels of PPARγ (*p* < 0.01), and reduced the expression of PGC-1α (*p*<0.01). These results suggest that the key factors for the therapeutic effect of serum are the incoming component of DM rather than the serum itself, and the possible involvement of PPARγ in the treatment of DM in serum, which is consistent with bioinformatics analysis and animal experimental investigations.

## 4 Discussion

The liver takes up a part in many physiological processes by regulating glucolipid metabolism throughout the body. In the context of MetS, IR and relative insulin deficiency result in increased lipolysis of adipose tissue, which increases fatty acid uptake by the liver, and hyperinsulinemia and IR will further increase lipid accumulation in the liver and increase the hepatic load. Thus, liver function is a key determinant of metabolic abnormalities. Due to the complex relationship between T2DM and NAFLD, the most current clinical therapies are not fully adequate for the treatment of complex diseases. Therefore, we are looking for a promising drug as a therapeutic approach. Chinese medicine has multi-target and multi-level characteristics, which provide unique superiority in the prophylaxis and therapy of complex diseases. Nevertheless, at the same time, due to the multiple and complex ingredients of DM, identifying them and describing their respective mechanisms in T2DM-NAFLD is a huge and complex project. In this research, the combination of network pharmacology and TMTs was used for predicting the active ingredients, therapeutic targets and important pathways in DM.

Identification of bioactive compounds is critical in studying mechanisms of action of complex herbal formulations. The network pharmacology analysis conducted in the present study identified varied bioactive that contribute mostly to the actions of DM including kaempferol, luteolin, baicalein, diosgenin, wogonin, quercetin, etc. Each of the bioactive compounds has been independently demonstrated to be effective against NAFLD based on cellular or animal studies, either by preventing the deterioration from simple steatosis to NASH or protecting against the “two hits” in NAFLD (Li et al., 2013; Chen et al., 2017; Zhu et al., 2018; Yang et al., 2019; Sun et al., 2020; Liu et al., 2021; Ochiai et al., 2021; Tie et al., 2021; Li et al., 2022; Zhou et al., 2022). Related studies of these active ingredients indicate that DM is indispensable in the treatment of T2DM-NAFLD. Therefore, our results first demonstrated the capability of DM in attenuating hepatic pathological changes in *db/db* mice as evidenced by reduced RBG, body weight, blood lipids, ransaminase, liver weight, lipid droplets, and lipid accumulation.

In this study, we network pharmacology and TMTs results for comparative analysis to further identify key genetic targets in the pathogenesis of NAFLD and T2DM. The results of network pharmacology pointed that AKT1, TNF, IL-6, PPARG and IL-1 β are the most critical core targets of DM for T2DM-NAFLD. The effect of DM on reducing inflammatory factors in diabetic rats and regulating Akt1 in diabetic mice has been confirmed in previous studies. In this study, In this study, protein changes in *db/db* mice after DM treatment as determined by TMT-based proteomics methods were focused. At the same time, go analysis of proteomics once again verified that DM was closely related to lipid metabolism, inflammation, and cytokine receptor binding, etc.

The leading KEGG pathway that linked these two diseases was the PPAR signaling pathway in diabetic complications by two bioinformatics. Analysis based on TMT shows that DM has significantly downregulated nine genes and upregulated two genes in the PPAR pathway. The downregulation of *plin2, scp2,* and *scd1* has demonstrated their positive involvement in improving lipid metabolism and reducing inflammation (Libby et al., 2018; Tao et al., 2020; Xu et al., 2022). In the past two decades, PPARγ has attracted much attention as a transcription factor regulating glucose and lipid homeostasis (Wang et al., 2020). Among the PPAR subtypes, PPARγ is mainly distributed in adipose tissue and can regulate the expression of target genes after being recognized, combined, and activated by endogenous and exogenous ligands. These target genes involved many biological effects such as cell energy metabolism, material metabolis, and cell proliferation. PPARγ agonists increased the sensitivity of peripheral tissues to insulin, including increased muscle uptake and utilization of glucose, inhibition of liver gluconeogenesis, inhibition of fatty acid decomposition and synthesis in adipose tissue, and promotion of adipocyte remodeling (Yessoufou and Wahli, 2010; Zhong et al., 2018; 2017). Therefore, PPARγ agonist as an insulin sensitizer is effective in the treatment of diabetes. Actually, whether some PPARγ agonists produce adverse reactions such as lipogenesis and edema depends on the intensity of PPARγ target gene regulation. Some agonists stimulate PPARγ activity, which can regulate the transcription of target genes related to therapeutic effects, but not the transcription of target genes related to adverse reactions. This is the fundamental reason why some agonists can retain therapeutic effects and avoid adverse reactions, its essence is the “selective” result of PPARγ activity regulation. PPARγ cofactors include coinhibitors and coactivators, the coactivators such as PPARγ coactivator one α(PGC-1α) and mediator complex subunit 1 (MED1), etc. PGC-1α is a key gene involved in regulating gluconeogenesis which plays an anti-diabetes role by inhibiting gluconeogenesis after being selectively inhibited (Kolli et al., 2014). The selective recruitment of cofactors determines the selectivity of PPARγ regulatory target genes. For instance, INT131 acts as an agonist of PPARγ but does not recruit MED1, which is a critical factor in regulating fat production. Therefore, INT131 can selectively reduce blood glucose without an obvious adverse reaction to fat production (Higgins and Mantzoros, 2008). The effect of DM was verified on this pathway. Our results suggested that DM effectively regulated the activated PPAR pathway, through the actions of upregulating the expressions of PPARγ, downregulating the expressions of PGC-1α, and reducing the inflammatory actions. A similar observation was obtained from our *in vitro* investigation. The serum containing DM obtained from SD rats was tested on the PA-induced HepG2 cells. Serum-containing DM reduced the lipids accumulation as shown by the oil red o staining. It also showed the regulatory effects on PPARγ and PGC-1α protein expressions. Taken together, our data suggested DM as a prospective therapy agent in treating NAFLD in T2DM, through selectively activating PPARγ without obvious adverse reaction.

## 5 Conclusion

Although the abilities of DM to orchestrate hepatic physiological processes have been discerned by modulating PPARγ and PGC1α, their specific contributions to T2DM with NAFLD remain unclear. In addition, we do not know if these bioactive compounds would be present in the serum and/or target organs at the contractions that could elicit meaningful pharmacological effects. Future studies are needed to solve the puzzles to advance our understanding the of functions of DM in the complex landscape of the human liver disease.

## Data Availability

The datasets presented in this study can be found in online repositories. The names of the repository/repositories and accession number(s) can be found below: ProteomeXchange Consortium (http://proteomecentral.proteomexchange.org) *via* the iProX partner repository with the dataset identifier PXD039727.
