# Supplementary material for: Dendrobium mixture ameliorates type 2 diabetes mellitus with non-alcoholic fatty liver disease through PPAR gamma: An integrated study of bioinformatics analysis and experimental verification
Source: Front Pharmacol. 2023 Feb 16;14:1112554. doi: 10.3389/fphar.2023.1112554 (PMC9978952; doi:10.3389/fphar.2023.1112554)
Supplement: Supplementary file 1 [file Image1.pdf]

Supplementary Material

Dendrobium mixture ameliorates non-alcoholic fatty liver disease induced by type 2 diabetes mellitus through PPAR gamma: an integrated study of bioinformatics analysis and experimental verification

Shuting Zhuang[1], Jieping Zhang[1*], Xiaohui Lin[1], Xiaoning Wang[1], Wenzhen Yu[1], Hong Shi[1*]

*** Correspondence:**

Jieping Zhang; Hong Shi
zhangjieping@fjtcm.edu.cn; shihong@fjtcm.edu.cn

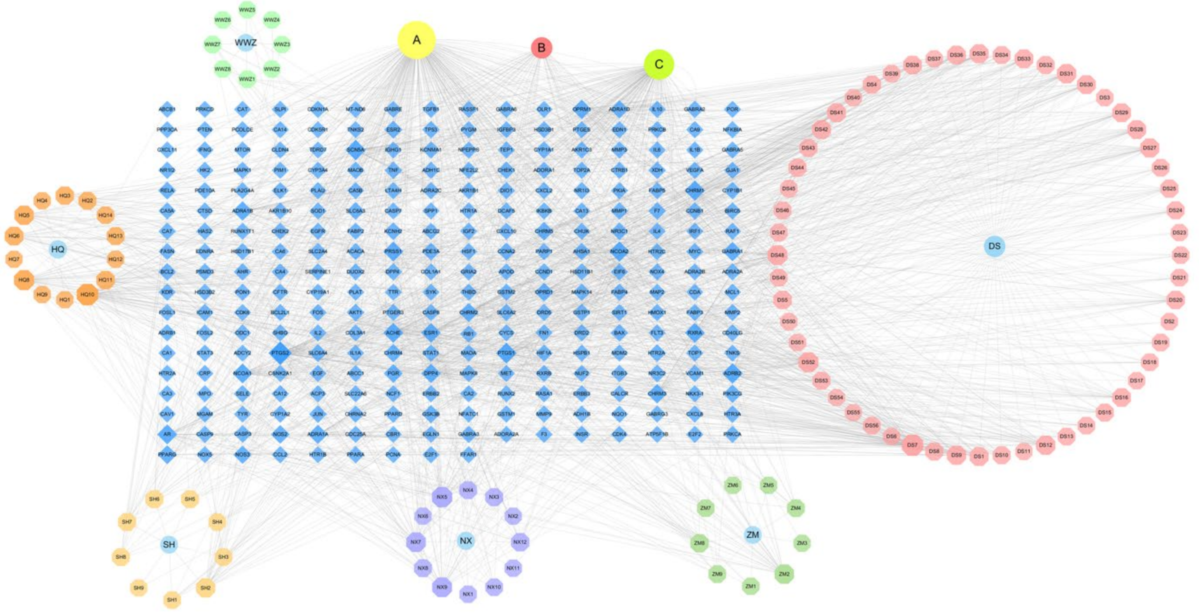

Supplementary Figure 1. Biological process, cellular component and molecular function relevant to the pharmacological action of DM. HQ: Huang Qi [Astragalus membranaceus (Fisch.) Bge.]; SH: Shi Hu (Dendrobium nobile Lindl.); WWZ: [Schisandra chinensis (Turcz.) Baill.]; NX: Niu Xi (Achyranthes bidentata Bl.); ZM: (Anemarrhena asphodeloides Bge.); DS: Dan Shen (Salvia miltiorrhiza Bge.); A: Querce-tin, B: Stigmasterol, C: Kaempferol.
